# Experimental Assessment of *Moringa oleifera* Leaf and Fruit for Its Antistress, Antioxidant, and Scavenging Potential Using *In Vitro* and *In Vivo* Assays

**DOI:** 10.1155/2012/519084

**Published:** 2011-12-14

**Authors:** Suaib Luqman, Suchita Srivastava, Ritesh Kumar, Anil Kumar Maurya, Debabrata Chanda

**Affiliations:** Molecular Bioprospection Department, Biotechnology Division, Central Institute of Medicinal and Aromatic Plants (Council of Scientific and Industrial Research), Lucknow 226015, India

## Abstract

We have investigated effect of *Moringa oleifera* leaf and fruit extracts on markers of oxidative stress, its toxicity evaluation, and correlation with antioxidant properties using *in vitro* and *in vitro* assays. The aqueous extract of leaf was able to increase the GSH and reduce MDA level in a concentration-dependent manner. The ethanolic extract of fruit showed highest phenolic content, strong reducing power and free radical scavenging capacity. The antioxidant capacity of ethanolic extract of both fruit and leaf was higher in the *in vitro* assay compared to aqueous extract which showed higher potential *in vivo*. Safety evaluation studies showed no toxicity of the extracts up to a dose of 100 mg/kg body weight. Our results support the potent antioxidant activity of aqueous and ethanolic extract of *Moringa oleifera* which adds one more positive attribute to its known pharmacological importance.

## 1. Introduction

*Moringa oleifera *Lam (syn. *M. pterygosperma*; commonly known as “The Miracle Tree,” “Horseradish-tree,” or “Ben oil tree”) is the best known and most widely distributed species of *Moringa*ceae family, having an impressive range of medicinal uses with high nutritional value throughout the world. Native to Western and sub-Himalayan tracts, India, Pakistan, Asia, and Africa [1, 2], this plant is well distributed in the Philippines, Cambodia, America, and the Caribbean Islands [3]. Organizations such as Trees for Life, Church World Service, and Educational Concerns for Hunger Organization have advocated *Moringa* as “Natural Nutrition for the Tropics” in various parts of the world [4–11]. Almost every part of this highly esteemed tree have long been consumed by humans and used for various domestic purposes as for alley cropping, animal forage, biogas, domestic cleaning agent, blue dye, fertilizer, foliar nutrient, green manure, gum (from tree trunks), honey and sugar cane juice-clarifier (powdered seeds), ornamental plantings, biopesticide, pulp, rope, tannin for tanning hides, water purification, machine lubrication (oil), manufacture of perfume, and hair care products [6].

Besides culinary and other domestic uses, several biological properties ascribed to various parts of this tree have been reviewed in the past [10, 12]. The leaves of *M. oleifera *have been reported to be a valuable source of both macro- and micronutrients, rich source of *β*-carotene, protein, vitamin C, calcium, and potassium and act as a good source of natural antioxidants; and thus enhance the shelf-life of fat-containing foods [7, 13]. Fruit (pod)/drum sticks and leaves have been used to combat malnutrition, especially among infants and nursing mothers for enhancing milk production [7, 14] and also regulate thyroid hormone imbalance [15–17].

A number of medicinal properties attributed to different parts of *Moringa* have been recognized by both Ayurvedic and Unani systems of medicines [2]. The plant finds its wide applicability in the treatment of cardiovascular diseases as the roots, leaves, gum, flowers, and infusion of seeds have nitrile, mustard oil glycosides, and thiocarbamate glycosides as their chemical constituents which are suggested to be responsible for the diuretic, cholesterol lowering, antiulcer, hepatoprotective, and cardiovascular protective property of the tree [10, 15, 16, 18–28]. The roots have been reported to possess antispasmodic activity through calcium channel blockade which forms the basis for its traditional use in diarrhoea [20, 29]. It also possesses antimicrobial activity due to its principle component pterygospermin. The fresh leaf juice was found to inhibit the growth of human pathogens as *Staphylococcus aureus *and *Pseudomonas aeruginosa *[30–33]. Phytoconstituents from different parts of the tree as niazimicin, niaiminin, various carbamates, and thiocarbamates (Table  1 Supplementry materials available at doi: 10.1155/2012/519048) have shown to exhibit antitumor activity *in vitro *[34–36]. The flowers show effective hepatoprotective effect due to the presence of quercetin [29–37]. Seeds are used as biosorbent for the removal of cadmium from aqueous medium and are one of the best-known natural coagulants discovered so far [38, 39]. They are also considered to be antipyretic, acrid, and bitter and reported to show antimicrobial activity [40, 41].

Various parts of the plant and their active constituents are known to possess diverse biological activity, however, little is known scientifically about the antioxidant potential of fruit (pod) and leaves of *Moringa oleifera*. Therefore, the present study investigates, establishes, and explains a comparative analysis of concentration- and dose-dependent effect of ethanolic and aqueous extract of *M. oleifera *leaves and fruit on markers of oxidative stress, its safety profile in mice model, and correlation with antioxidant properties using *in vitro *and *in vivo *assays.

## 2. Materials and Methods

### 2.1. Chemicals

All the chemicals, reagents, and solvents used in the assay protocols were of analytical grade. Ascorbic acid, sodium phosphate, phosphate buffer saline, thiobarbituric acid, ammonium molybdate, ethyldimine tetraacetic acid (EDTA), sodium citrate, DPPH, DMSO, Folin-Ciocalteau reagent, sodium acetate trihydrate, sodium hydroxide tricarboxylic acid, and sodium citrate were obtained from Sigma Aldrich, India. Other chemicals such as gallic acid, TRIS-HCl, sodium carbonate, sodium chloride, potassium ferricynide, ferric chloride, tripyridyl-s-triazine (TPTZ), potassium dihydrogen phosphate, disodium hydrogen phosphate, metaphosphoric acid, and 5,5′-Dithiobis 2-nitro benzoic acid (DTNB) were purchased from Himedia, India. The solvents, methanol, ethanol, glacial acetic acid, sulphuric acid, hydrochloric acid, kits for determination of RBC, WBC and haemoglobin, activity kits for alkaline Phosphatase (ALKP), serum glutamic pyruvic transaminase (SGPT), and serum glutamic oxaloacetic transaminase (SGOT) were ordered from E-Merck Ltd., India.

### 2.2. Collection of Plant Material and Preparation of Extract

The leaves and fruit (pod) of *Moringa oleifera *Lam (syn. *M. pterygosperma*) were collected from the research farm of the Central Institute of Medicinal and Aromatic Plants (CSIR), Lucknow, India, and the herbarium specimens were deposited in Gyan Surabhi of CIMAP, Lucknow (Voucher Specimen no. 9076). Plant material was authenticated by Drs. A. K. Gupta, S. C. Singh, S. P. Jain, and J. Singh. The leaves and fruit (pod) samples were washed properly and dried at 40°C. The dried samples were extracted with 100% ethanol and distilled water. The extracts were collected at least three times and were filtered through Whatman number 1 filter paper and then concentrated on rotary evaporator (Buchi, Flavil, Switzerland) at 45°C, dried and kept at 4°C till used for the assay. The sample and solvent mass ratio was 1 : 2 during extraction. The extracts were dissolved/solubilised in DMSO and diluted with sterile water to get the final concentration as per requirement.

### 2.3. Selection of Animals and Dose Administration

In view of potent antioxidant activity of *M. oleifera *aqueous and ethanolic extracts in *in vitro *model, acute oral toxicity of the same was carried out in Swiss albino mice for its further development into drug product. Experiment was conducted in accordance with the Organization for Economic Co-operation and Development (OECD) test guideline number 423 (1987).

For the study, 24 mice were taken and divided into six groups comprising male mice in each group weighing between 25 and 30 g. The animals were maintained at 22 ± 5°C with humidity control and also on an automatic dark and light cycle of 12 hours. The animals were fed with the standard rat feed and provided *ad libitum *drinking water. Mice of group 1 were kept as control and animals of groups 2, 3, 4, 5, and 6 were kept as experimental. The animals were acclimatized for 7 days in the experimental environment prior to the actual experimentation. The test extracts were given at 0.01, 0.1, 1, 10, and 100 mg/kg body weight to animals of groups 2, 3, 4, 5, and 6, respectively. Control animals received only vehicle. All the animals were sacrificed on the seventh day after the experimentation.

### 2.4. Collection of Blood and Serum and Isolation of Packed Erythrocytes

Retino-orbital blood from healthy mice (*Mus musculus*) was collected for experiments using Heparin (10 units/mL) as the anticoagulant from all the animals on the seventh day after experiment. The collected blood was stored at 4°C and was used for experiments within four hours of collection. The serum samples were stored at −20°C and were used within 2 days of collection [42–44]. The blood samples were centrifuged at 4°C for 10 min at 3000 rpm to remove plasma and buffy coat. The isolated erythrocytes were washed with 0.154 mol/L normal saline (NaCl) thrice for packed erythrocytes.

### 2.5. *In Vitro* Experiments

#### 2.5.1. Total Phenolic Estimation

The total phenolic content of the aqueous and ethanolic extract of *M. oleifera *was estimated in terms of gallic acid equivalence by Folin-Ciocalteau reagent by the method of Singleton and Rossi [45], with slight modification [46]. The Folin-Ciocalteau assay relies on the transfer of electrons in alkaline medium from phenolic compounds to phosphomolybdic/phosphotungstic acid complexes, which are determined spectroscopically at 765 nm. Different concentration of aqueous and ethanolic extracts of *M. oliefera *extracts (10, 50, 100, 250, 500, and 1000 *μ*g/mL) was mixed with Folin's reagent (1 : 9 in distilled water) and 7.5% Na_2_CO_3_ and incubated at 37°C for 90 min. The absorbance was measured at 765 nm.

#### 2.5.2. Reducing Power Assay

The reducing power of the aqueous and ethanolic extract of *M. oleifera *was determined by the method of Yen and Chen [47] with slight modification reported previously [46]. Briefly, for *in vitro *analyses, different concentrations of extract (10, 50, 100, 250, 500, and 1000 *μ*g/mL) was mixed with 0.2 M phosphate buffer (pH 6.6) and 1% K_4_FeCN_6_ and incubated for 20 min at 50°C followed by precipitation with 10% TCA. The supernatant was diluted with equal volume of distilled water and ferric reducing capacities of the extracts were checked by adding 0.10% FeCl_3_. The absorbance was recorded at 700 nm against a reagent blank.

#### 2.5.3. FRAP Assay

The ferric reducing antioxidant power (FRAP) measures the antioxidant capacity to reduce the Fe^+++^/tripyridyl-s-triazine (TPTZ) complex, to the ferrous form [46, 48]. The FRAP reagent was freshly prepared by mixing 10 mM TPTZ with 20 mM ferric chloride in 300 mM acetate buffer, pH 3.6 in the ratio 1 : 1 : 10. Different concentrations (10, 50, 100, 250, 500, and 1000 *μ*g/mL) of aqueous and ethanolic extract of *M. oleifera *were added and the decrease in the absorption by the complex was measured after 5 min at 593 nm at room temperature. The activities were calculated by comparing the concentration of each extract with the concentration of Fe^++^ required to give the same absorbance change using FeSO_4_ as standard.

#### 2.5.4. DPPH Assay

Radical scavenging activity of aqueous and ethanolic extract of *M. oleifera *was measured by the modified DPPH method [46, 49]. DPPH in ethanol is a stable radical, dark violet in color. Its color is bleached by its reaction with a hydrogen donor. For *in vitro *analyses, various concentrations of each extracts (10, 50, 100, 250, 500, and 1000 *μ*g/mL) were added to 100 mM TRIS-HCl buffer (pH 7.4) and 100 *μ*M of DPPH. The reaction mixture was incubated for 30 min in the dark at room temperature and measured at 515 nm, against a blank.

#### 2.5.5. Total Antioxidant Capacity Estimation

The total antioxidant capacity of *M. oleifera *was evaluated by the method described in [50]. The total antioxidant capacity of various concentrations of ethanolic and aqueous extracts of *M. oleifera *(10, 50, 100, 250, 500, and 1000 *μ*g/mL) was determined with phosphomolybdenum using ascorbic acid as the standard in 1 mL of TAC reagent (3.3 mL sulphuric acid, 335 mg sodium phosphate, and 78.416 mg ammonium molybdate in 100 mL of distilled water). The sample mixtures were incubated in a boiling water bath at 95°C for 90 min. The absorbance of the samples was measured at 695 nm. The blank solution contained 1.0 mL of reagent solution and the appropriate volume of the same solvent used for the sample.

### 2.6. *In Vivo * Experiments

#### 2.6.1. Estimation of Reduced Glutathione Concentration

The reduced glutathione concentration in erythrocytes was estimated using standard method of Beutler [51], as reported previously [44] with slight modification. To 100 *μ*L of packed RBC, 900 *μ*L of phosphate solution was added. Tubes were centrifuged at 5000 rpm for 5 min and supernatant was discarded. To 100 *μ*L of RBC (pellet), 100 *μ*L of phosphate solution was added. Out of 200 *μ*L of cell suspension, to 100 *μ*L of it, 900 *μ*L of distilled water was added followed by the addition of 1.5 mL precipitation solution and tubes were centrifuged at 5000 rpm for 3 min. To the 50 *μ*L of supernatant, 200 *μ*L phosphate solutions and 25 *μ*L of freshly prepared DTNB were added. This method is based on the ability of the sulfhydryl group to reduce 5,5′-Dithiobis 2-nitro benzoic acid (DTNB) and form a yellow-colored anionic product whose absorbance is measured at 412 nm. Concentration of GSH is expressed as *μ*mol/mL of packed erythrocytes and was determined from a standard plot.

#### 2.6.2. Determination of Malondialdehyde Concentration

Erythrocyte malondialdehyde formed during lipid peroxidation was measured according to the method of Esterbauer and Cheeseman [52], as described earlier [44]. Packed erythrocytes (200 *μ*L) were suspended in 3 mL of PBS-glucose solution (pH 7.4). To 1.0 mL of the suspension, 1.0 mL of 10% TCA was added. Centrifugation was done for 5 min at 5000 rpm. To 1.0 mL of supernatant, 1.0 mL of 0.67% TBA in 0.05 mol/L NaOH was added. Tubes were kept in boiling water bath for 20 min at temperature greater than 90°C and cooled. Absorbance was measured at 532 nm (OD_1_) and 600 nm (OD_2_) against a blank. The net optical density (OD) was calculated after subtracting absorbance at OD_2_ from that of OD_1_. The concentration of MDA was determined from a standard plot and expressed as nmol/mL of packed erythrocytes. 

#### 2.6.3. Total Phenolic Estimation

The serum samples were taken from 6 groups of mice administrated with different doses (0.01, 0.1, 1, 10, 100 mg/kg b/w) of aqueous and ethanolic extracts of *M. oliefera*, along with a control group and was mixed with Folin's reagent (1 : 9 in distilled water) and 7.5% Na_2_CO_3_. Samples were incubated at 37°C for 90 min. The absorbance was measured at 765 nm against the reference blank. The serum samples from mice administered with gallic acid was used as the standard reference (Supplementary material).

#### 2.6.4. Reducing Power Assay

The reducing power of the serum samples of mice administrated with different doses (0.01, 0.1, 1, 10, 100 mg/kg b/w) of aqueous and ethanol extracts of *M. oliefera*, along with a control were analyzed by mixing serum, with 0.2 M phosphate buffer (pH 6.6) and 1% K_4_FeCN_6_ and incubated for 20 min at 50°C followed by precipitation with 10% TCA. The supernatant was diluted with equal volume of distilled water and ferric reducing capacities of the extracts were checked by adding 0.10% FeCl_3_. The absorbance was recorded at 700 nm against a reagent blank.

#### 2.6.5. FRAP Assay

The serum samples of all the six groups of mice were added with FRAP reagent and the decrease in the absorption by the complex was measured after 5 min at 593 nm at room temperature. The activities were calculated by comparing the concentration of each extract with the concentration of Fe^++^ required to give the same absorbance change using FeSO_4_ as standard.

#### 2.6.6. DPPH Assay

For *in vivo *analyses, serums of mice mice administrated with different doses (0.01, 0.1, 1, 10, 100 mg/kg b/w) of aqueous and ethanolic extracts of *M. oliefera*, were added to 100 mM TRIS-HCl buffer (pH 7.4) and 100 *μ*M of DPPH. The reaction mixture was incubated for 30 min in the dark at room temperature and measured at 515 nm, against a reference blank. Serum taken from mice administered with Ascorbic acid and Gallic acid were used as standard.

#### 2.6.7. Total Antioxidant Capacity Estimation

The *in vivo *analyses were performed by mixing serum samples from mice of different groups in 1 mL of TAC reagent and incubating the sample mixtures in a boiling water bath at 95°C for 90 min. The absorbance of the samples was measured at 695 nm. The serum samples from mice administered with ascorbic acid was used as the standard reference (Supplementary material).

### 2.7. Observational, Haematological, Biochemical, and Gross Pathological Study

The animals were checked for mortality and any sign of ill health at hourly interval on the day of administration of drug and there after a daily general case side clinical examination was carried out including changes in skin, mucous membrane, eyes, occurrence of secretion and excretion, and also responses like lachrymation, pilo-erection respiratory patterns, and so forth. Also changes in gait, posture, and response to handling were also recorded [53]. In addition to observational study, body weights were recorded and blood and serum samples were collected from all the animals on the seventh day after experiment and were analysed for total RBC, WBC, haemoglobin percentage, and biochemical parameters like ALKP, SGPT and SGOT activity using kits from E-Merck Ltd. in a semi-autoanalyser (RA-50) made by Bayer Co Ltd., Germany [54].

### 2.8. Statistical Analysis

The data were subjected to two- tailed Student's “*t*” test with Welch correction and linear regression analysis using Graph Pad InStat 3.06 Version. Values expressed are mean of three replicate determinations ± standard deviation.

## 3. Result

A concentration- and dose-dependent *in vitro *and *in vivo *evaluation of the oxidative stress biomarkers, toxicity markers, and its correlation with the antioxidative properties was studied for the ethanolic and aqueous extracts of fruit and leaves. Activity of extracts were tested by performing *in vitro *and *in vivo *assays, namely: GSH, MDA, FRAP, DPPH, TPC, RP, TAC, SGPT, SGOT, ALKP, Hb, WBC, RBC, and body weight determination and observations are presented in Figures 1–19.

### 3.1. *In Vitro* Analysis

#### 3.1.1. Total Phenolic Content

**Figure 1:**
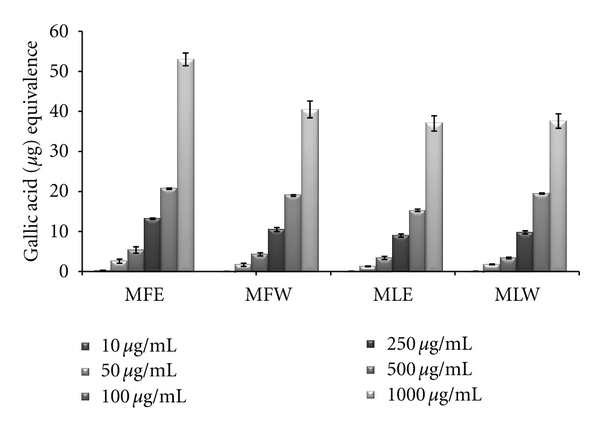
Concentration-dependent total phenolic estimation of ethanolic and aqueous extract of *Moringa oleifera *fruit and leaf (MLW: *Moringa* leaf aqueous extract, MLE: *Moringa* leaf ethanol extract, MFW: *Moringa* fruit (pod) aqueous extract, MFE: *Moringa* fruit (pod) ethanol extract; values are mean ± SD of three independent experiments in replicates at each concentration and estimated in terms of gallic acid equivalence).

Plant phenolics constitute one of the major groups of compounds acting as primary antioxidants or free radical terminators; it was worth determining their total amount (TPC) in the leaves and fruit extract of *M. oleifera*. Total phenolic content increased in the concentration-dependent manner having a gallic acid equivalent of 22 ± 2 *μ*g/mL for all the extracts. But there is significant rise in the phenolic content of fruit ethanolic (MFE) extract 53 ± 1.8 *μ*g/mL GAE (Figure 1). A significant pattern (*P* = 0.0053) was observed both in the *in vitro *studies and *in vivo *analysis.

#### 3.1.2. Reducing Power

**Figure 2:**
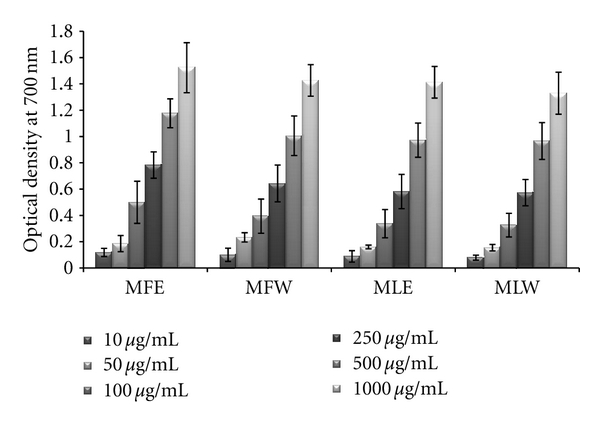
Concentration-dependent reducing power of ethanolic and aqueous extract of *Moringa oleifera *fruit and leaf (MLW: *Moringa* leaf aqueous extract, MLE: *Moringa* leaf ethanol extract, MFW: *Moringa* fruit (pod) aqueous extract, MFE: *Moringa* fruit (pod) ethanol extract; values are mean ± SD of three independent experiments in replicates at each concentration).

The extracts showed an increasing trend in reducing power as the concentration rises. The extract MFE (*P* < 0.0001) has the highest reducing power capacity (Figure 2) among all the four extracts following the trend MFE > MFW ~ MLE > MLW.

#### 3.1.3. DPPH

**Figure 3:**
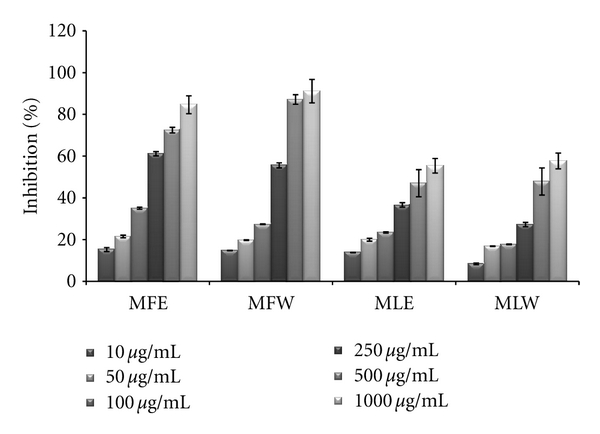
Concentration-dependent free radical scavenging (DPPH) activity of ethanolic and aqueous extract of *Moringa oleifera *fruit and leaf (MLW: *Moringa* leaf aqueous extract, MLE: *Moringa* leaf ethanol extract, MFW: *Moringa* fruit (pod) aqueous extract, MFE: *Moringa* fruit (pod) ethanol extract; values are mean ± SD of three independent experiments in replicates at each concentration).

The scavenging activity of the DPPH radical was tested by reduction of the stable radical DPPH to the yellow-colored diphenylpicrylhydrazine. The experimental observations of scavenging effect of *M. oleifera *extracts with the DPPH radical are depicted in Figure 3. *In vitro *analysis reveals that fruit extract (MFE~MFW > MLE~MLW) was better scavengers than the leaves (*P* < 0.001). With an increase in the concentration, the scavenging potential of the extracts increased by combating formation of the DPPH free radical.

#### 3.1.4. FRAP

**Figure 4:**
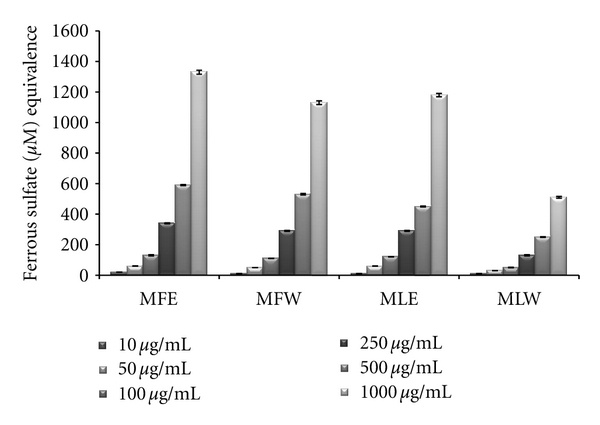
Concentration-dependent ferric reducing antioxidant power of ethanolic and aqueous extract of *Moringa oleifera *fruit and leaf (MLW: *Moringa* leaf aqueous extract, MLE: *Moringa* leaf ethanol extract, MFW: *Moringa* fruit (pod) aqueous extract, MFE: *Moringa* fruit (pod) ethanol extract; values are mean ± SD of three independent experiments in replicates at each concentration and expressed in terms of ferrous sulphate equivalence).

Ferric reducing antioxidant power of the extract of *M. oleifera *fruit and leaves was expressed as equivalence of ferrous sulphate (*μ*M/L) and our observation depicts that the ferric reducing antioxidant power of the extracts was in the increasing trend with the concentrations (Figure 4). The observations of FRAP analysis also showed a positive correlation with results of the reducing power and DPPH radical scavenging analysis. The ethanolic extract of fruit (MFE; *P* < 0.0001) showed all the three activities with highest efficiencies followed by the other three extracts (MFW, MLE, and MLW).

#### 3.1.5. Total Antioxidant Capacity

**Figure 5:**
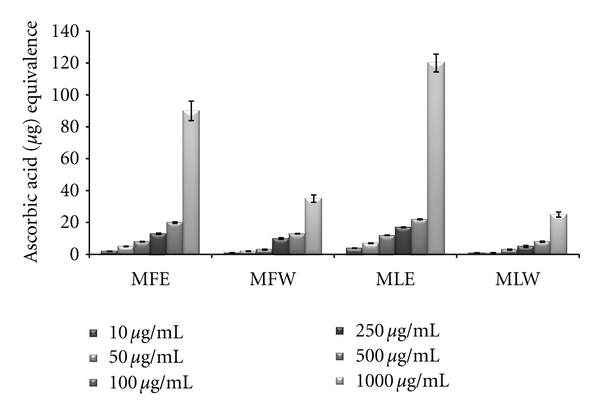
Concentration-dependent total antioxidant capacity of ethanolic and aqueous extract of *Moringa oleifera *fruit and leaf (MLW: *Moringa* leaf aqueous extract, MLE: *Moringa* leaf ethanol extract, MFW: *Moringa* fruit (pod) aqueous extract, MFE: *Moringa* fruit (pod) ethanol extract; values are mean ± SD of three independent experiments in replicates at each concentration and determined in terms of ascorbic acid equivalence).

The total antioxidant capacity of the extracts was determined in terms of ascorbic acid equivalence, and our result suggests MLE (*P* < 0.001) exhibits the highest antioxidant capacity (MLE > MFE > MFW > MLW). The antioxidant capacity of the extracts increases with increase in their concentration (Figure 5).

### 3.2. *In Vivo * Analysis

The effect on the biomarkers of oxidative stress was studied by evaluating the content of biomarkers GSH and MDA in mice erythrocytes.

#### 3.2.1. Reduced Glutathione and Malondialdehyde Concentration

**Figure 6:**
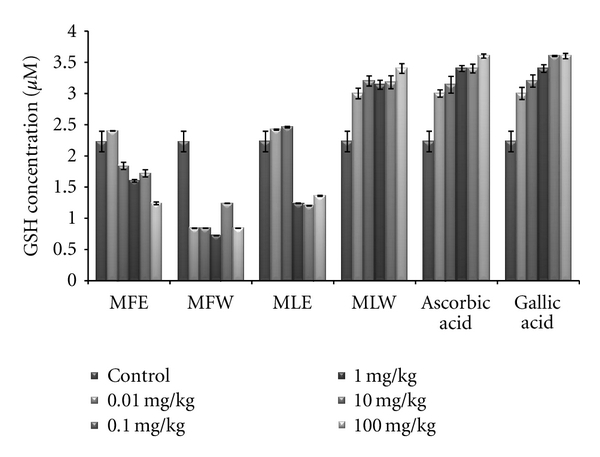
Effect of ethanolic and aqueous extract of *Moringa oleifera *fruit and leaf on reduced glutathione concentration of mice erythrocytes (MLW: *Moringa* leaf aqueous extract, MLE: *Moringa* leaf ethanol extract, MFW: *Moringa* fruit (pod) aqueous extract, MFE: *Moringa* fruit (pod) ethanol extract; values are mean ± SD of erythrocytes GSH content from replicates of six mice in each group at each dose).

**Figure 7:**
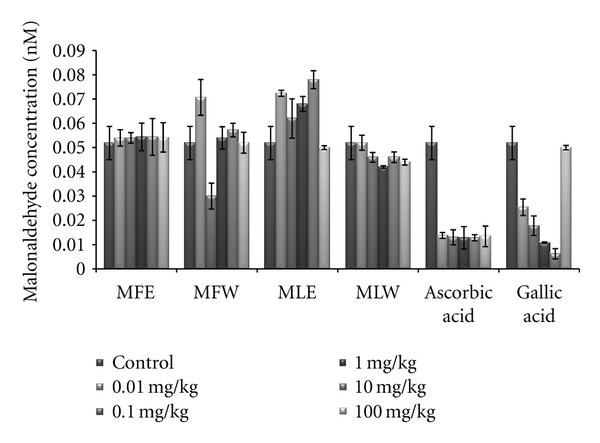
Effect of ethanolic and aqueous extract of *Moringa oleifera *fruit and leaf on malondialdehyde content of mice erythrocytes (MLW: *Moringa* leaf aqueous extract, MLE: *Moringa* leaf ethanol extract, MFW: *Moringa* fruit (pod) aqueous extract, MFE: *Moringa* fruit (pod) ethanol extract; values are mean ± SD of erythrocytes MDA content taken from replicates of six mice in each group at each dose).

Figure 6 shows the dose-responsive effect of alcoholic and aqueous extract of *Moringa* fruit and leaves on GSH content. Maximum enhancement of GSH was observed in mice erythrocytes treated with the aqueous extract of *Moringa* leaves (MLW). This rise is consistent with the increase in the dose of the extract. In our study, the significant increase of GSH content with the administration of aqueous extract of leaves in dose-dependent manner shows higher antioxidant capacity, compared to fruit extract (two tailed *t*-test, *P* = 0.0004). Similarly, the dose-responsive effect on lipid peroxidation was also observed (Figure 7). The basal value of MDA content was maintained in case of the aqueous extract of moringa leaf (MLW), compared to ethanolic extract where a rise in MDA content was recorded. The aqueous fruit extract (MFW) was able to lower down the MDA level significantly (*P* = 0.0121 for unpaired *t*-test) at 0.1 mg/kg b/w which again rose to normal level at higher concentrations.

**Figure 8:**
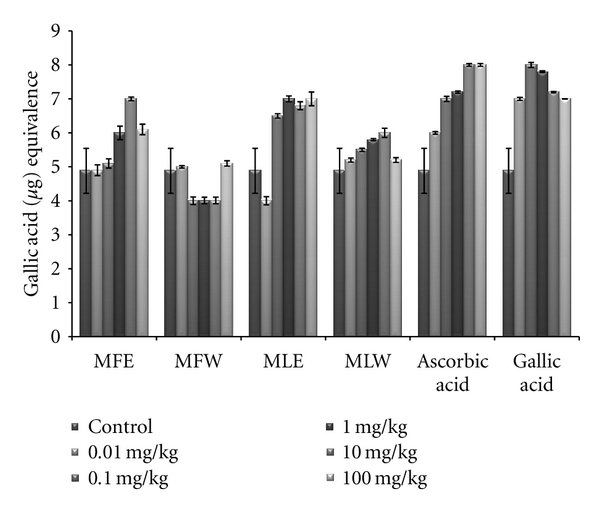
Dose-dependent total phenolic estimation of ethanolic and aqueous extract of *Moringa oleifera *fruit and leaf in mice serum (MLW: *Moringa* leaf aqueous extract, MLE: *Moringa* leaf ethanol extract, MFW: *Moringa* fruit (pod) aqueous extract, MFE: *Moringa* fruit (pod) ethanol extract; values are mean ± SD of serum taken from replicates of six mice in each group at each dose and estimated in terms of gallic acid equivalence).

**Figure 9:**
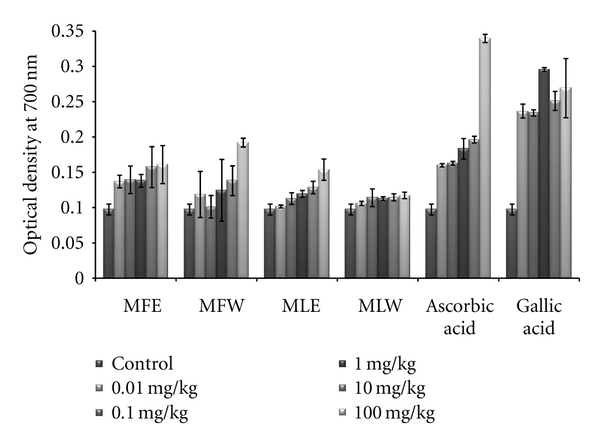
Dose-dependent reducing power estimation of ethanolic and aqueous extract of *Moringa oleifera *fruit and leaf in mice serum (MLW: *Moringa* leaf aqueous extract, MLE: *Moringa* leaf ethanol extract, MFW: *Moringa* fruit (pod) aqueous extract, MFE: *Moringa* fruit (pod) ethanol extract; values are mean ± SD of serum taken from replicates of six mice in each group at each dose).

**Figure 10:**
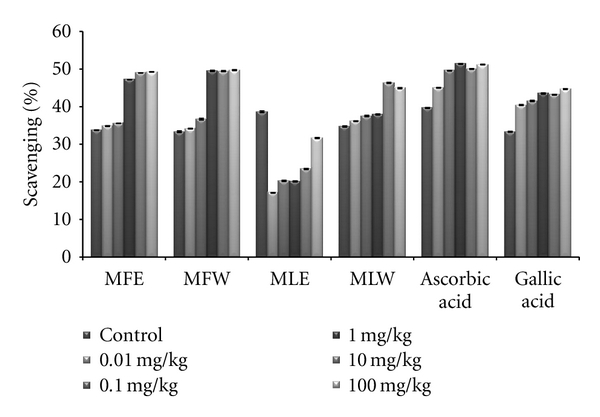
Dose-dependent free radical scavenging (DPPH) activity of ethanolic and aqueous extract of *Moringa oleifera *fruit and leaf in mice serum (MLW: *Moringa* leaf aqueous extract, MLE: *Moringa* leaf ethanol extract, MFW: *Moringa* fruit (pod) aqueous extract, MFE: *Moringa* fruit (pod) ethanol extract; Values are mean ± SD of serum taken from replicates of six mice in each group at each dose).

**Figure 11:**
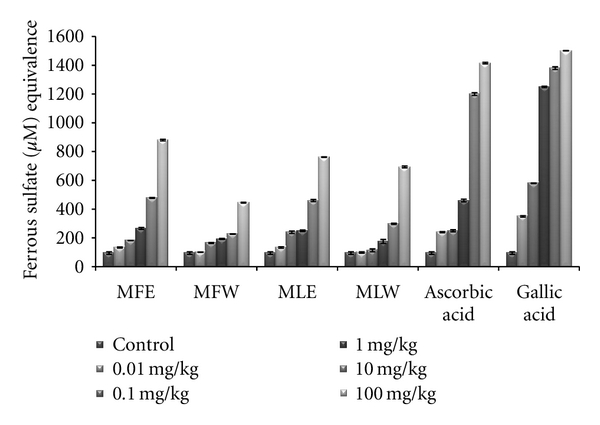
Dose-dependent ferric reducing antioxidant power of ethanolic and aqueous extract of *Moringa oleifera *fruit and leaf in mice serum (MLW: *Moringa* leaf aqueous extract, MLE: *Moringa* leaf ethanol extract, MFW: *Moringa* fruit (pod) aqueous extract, MFE: *Moringa* fruit (pod) ethanol extract; values are mean ± SD of serum taken from replicates of six mice in each group at each dose and expressed in terms of ferrous sulphate equivalence).

**Figure 12:**
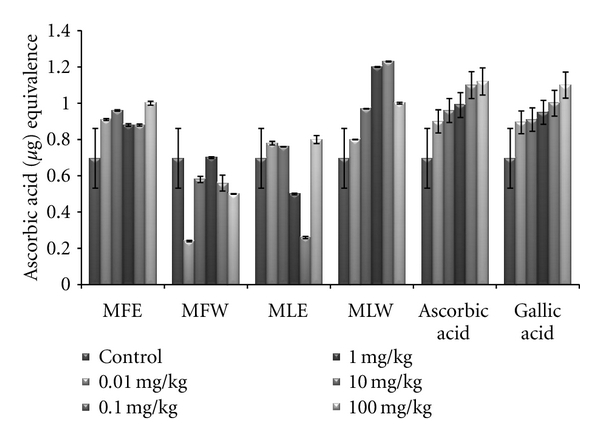
Dose-dependent total antioxidant capacity of ethanolic and aqueous extract of *Moringa oleifera *fruit and leaf in mice serum (MLW: *Moringa* leaf aqueous extract, MLE: *Moringa* leaf ethanol extract, MFW: *Moringa* fruit (pod) aqueous extract, MFE: *Moringa* fruit (pod) ethanol extract; values are mean ± SD of serum taken from replicates of six mice in each group at each dose and determined in terms of ascorbic acid equivalence).

To assess the correlation with antioxidative properties, total phenolic content, reducing power, ferric reducing, free radical scavenging, and total antioxidant capacity of the extracts were determined in serum samples of mice (Figures 8–12).

#### 3.2.2. Total Phenolic Content, Reducing Power, DPPH, FRAP, and Total Antioxidant Capacity

In the *in vivo *analysis of the extracts, the serum sample of mice administered with extract MFE showed the highest content of total phenolics at the dose of 10 mg/kg b/w congruent to the ethanolic extract of leaves (MLE; Figure 8). Increasing the dose showed no significant changes in the total phenolic content. The *in vivo* analysis of reducing power for the serum of mice administered with different doses of *Moringa oleifera* leaves and fruit extracts in dose-dependent manner showed conformity with that of *in vitro* analysis (MFE > MLE > MFW ~ MLW; Figure 9). Similarly, DPPH scavenging assay showed the same pattern (MFE ~ MFW > MLE ~ MLW) to that of *in vitro* analysis but the scavenging potential was observed to a dose of 1 mg/kg b/w after which, the effect was almost constant (Figure 10). In dose-dependent studies, the serum from mice groups administered with ethanolic extracts showed more ferric reducing antioxidant power than the serum of mice administered with aqueous extracts. The trend observed was MFE > MLE > MLW > MFW (Figure 11), whereas MLW showed highest antioxidant capacity (Figure 12). A linear correlation between DPPH radical scavenging activity, FRAP, and polyphenolic content (*P* < 0.0001) of the extract has been observed.

#### 3.2.3. Observational, Haematological, Biochemical, and Gross Pathological Analysis

**Figure 13:**
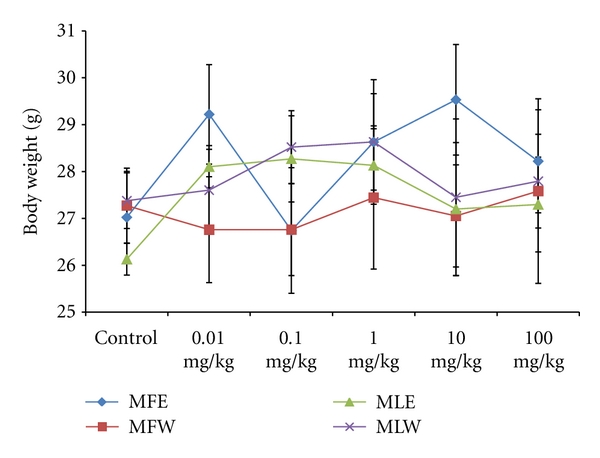
Dose-dependent effect of ethanolic and aqueous extract of *Moringa oleifera *fruit and leaf on body weight of mice (MLW: *Moringa* leaf aqueous extract, MLE: *Moringa* leaf ethanol extract, MFW: *Moringa* fruit (pod) aqueous extract, MFE: *Moringa* fruit (pod) ethanol extract; values are mean ± SD of serum taken from replicates of six mice in each group at each dose).

**Figure 14:**
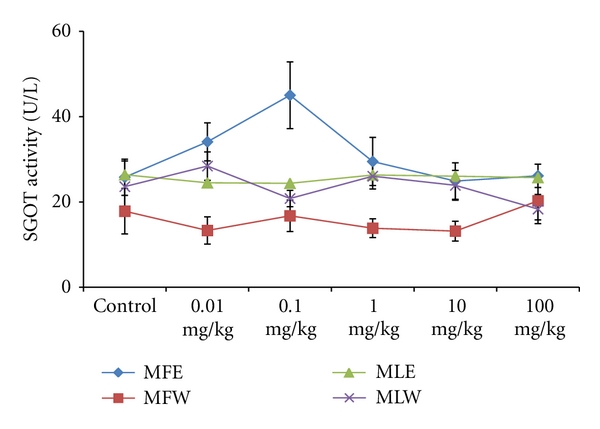
Dose-dependent effect of ethanolic and aqueous extract of *Moringa oleifera *fruit and leaf on SGOT activity of mice (MLW: *Moringa* leaf aqueous extract, MLE: *Moringa* leaf ethanol extract, MFW: *Moringa* fruit (pod) aqueous extract, MFE: *Moringa* fruit (pod) ethanol extract; values are mean ± SD of serum taken from replicates of six mice in each group at each dose).

**Figure 15:**
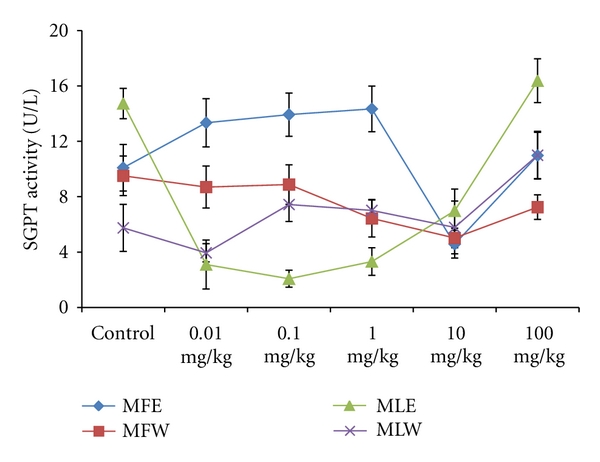
Dose-dependent effect of ethanolic and aqueous extract of *Moringa oleifera *fruit and leaf on SGPT activity of mice (MLW: *Moringa* leaf aqueous extract, MLE: *Moringa* leaf ethanol extract, MFW: *Moringa* fruit (pod) aqueous extract, MFE: *Moringa* fruit (pod) ethanol extract; values are mean ± SD of serum taken from replicates of six mice in each group at each dose).

**Figure 16:**
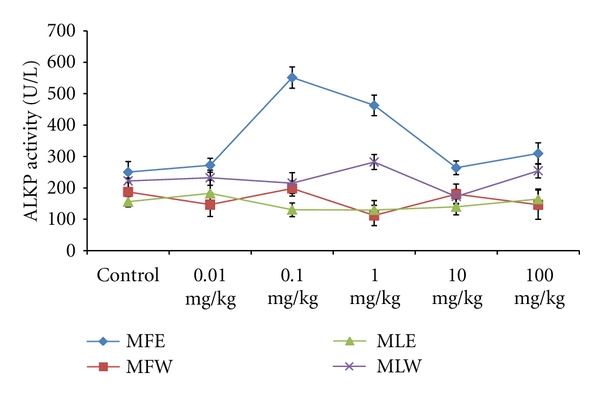
Dose-dependent effect of ethanolic and aqueous extract of *Moringa oleifera *fruit and leaf on ALKP activity of mice (MLW: *Moringa* leaf aqueous extract, MLE: *Moringa* leaf ethanol extract, MFW: *Moringa* fruit (pod) aqueous extract, MFE: *Moringa* fruit (pod) ethanol extract; values are mean ± SD of serum taken from replicates of six mice in each group at each dose).

**Figure 17:**
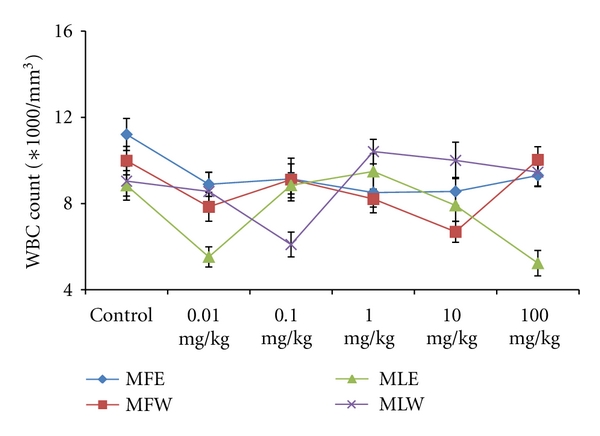
Dose-dependent effect of ethanolic and aqueous extract of *Moringa oleifera *fruit and leaf on WBC count of mice blood (MLW: *Moringa* leaf aqueous extract, MLE: *Moringa* leaf ethanol extract, MFW: *Moringa* fruit (pod) aqueous extract, MFE: *Moringa* fruit (pod) ethanol extract; values are mean ± SD of serum taken from replicates of six mice in each group at each dose).

**Figure 18:**
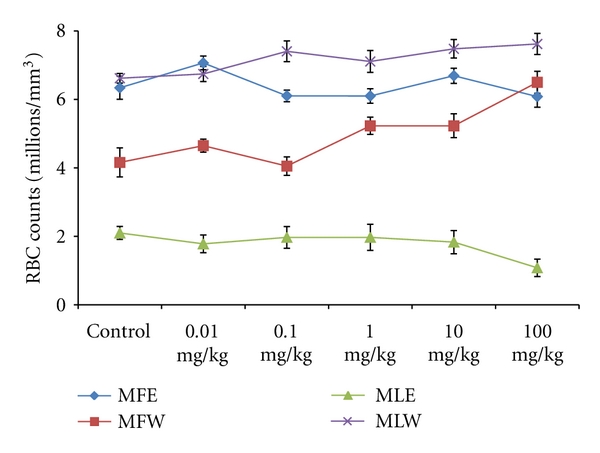
Dose-dependent effect of ethanolic and aqueous extract of *Moringa oleifera *fruit and leaf on RBC count of mice blood (MLW: *Moringa* leaf aqueous extract, MLE: *Moringa* leaf ethanol extract, MFW: *Moringa* fruit (pod) aqueous extract, MFE: *Moringa* fruit (pod) ethanol extract; values are mean ± SD of serum taken from replicates of six mice in each group at each dose).

**Figure 19:**
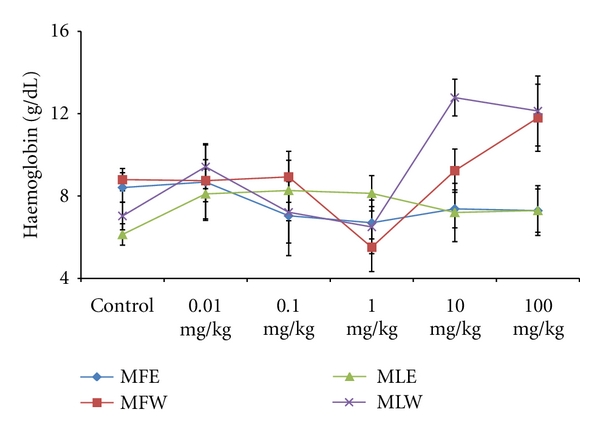
Dose-dependent effect of ethanolic and aqueous extract of *Moringa oleifera *fruit and leaf on haemoglobin level of mice blood (MLW: *Moringa* leaf aqueous extract, MLE: *Moringa* leaf ethanol extract, MFW: *Moringa* fruit (pod) aqueous extract, MFE: *Moringa* fruit (pod) ethanol extract; values are mean ± SD of serum taken from replicates of six mice in each group at each dose).

To study the toxicity of ethanolic and aqueous extract of *M. oleifera *leaves and fruit, animals were challenged with different doses and their safety profiles were determined by evaluating haematological, biochemical, and pathological parameters. The body weights recorded from all the animals on the seventh day after experiment were within 27 ± 1 g and showed no significant alterations (Figure 13). The biochemical parameters ALKP, SGPT, and SGOT showed nonsignificant changes, even increase in the dose of the extract was well tolerated and nontoxic (Figures 14, 15, and 16). The haematological parameters RBC, WBC, Hb were also analyzed (Figures 17, 18, and 19). Except total RBC count in MLE, no change was observed among the different extracts in the haematological studies. Even the value of RBC count is similar to that of vehicle control studied with same extract.

## 4. Discussion

For hundreds of years, traditional healers have prescribed different parts of *M. oleifera *for treatment of skin diseases, respiratory illnesses, ear and dental infections, hypertension, diabetes, cancer treatment, and water purification, and they have promoted its use as a nutrient dense food source [6, 10, 12]. Herein, we report a comparative *in vitro *and *in vivo *analysis of the effect of aqueous and ethanolic extracts of *Moringa* leaf and fruit on the markers of oxidative stress. Biomarkers of oxidative stress reflect environmental pro-oxidant and antioxidant ratio and also serve as a surrogate measure of a disease process. The protective effects of aqueous and ethanolic extract of *Moringa* leaf and fruit on erythrocyte GSH and MDA concentration may be attributed to the presence of phytoconstituents (polyphenols, tannins, anthocyanin, glycosides, thiocarbamates) that scavenge free radicals, activate the antioxidant enzymes, and inhibit oxidases [55, 56]. In present study, we studied both concentration- and dose-dependent analysis by estimating GSH and MDA concentration *in vitro *as well as *in vivo*.

Glutathione (GSH) acts as an antioxidant both intracellularly and extracellulary, and it is a major nonprotein sulfhydryl compound, with many biological functions, including maintenance of membrane protein-SH groups in the reduced form, the oxidation of which can otherwise cause altered cellular structure and function [57, 58]. Membrane-SH group oxidative damage may be an important molecular mechanism inducing changes in the membrane microelasticity or whole cell deformability of erythrocytes under conditions of physiological and pathological oxidative stress. The aqueous extract of *Moringa* leaves contains certain nonphenolic, biologically active components such as selenium, thiocarbamates, glucosinolates, its hydrolysis products as glucoraphanin, isothiocyanate sulforaphane, nitriles [22, 23], in addition to the phenolics, which could serve as antioxidants and may effectively scavenge various reactive oxygen species/free radicals under *in vivo *conditions. The ethanolic extract of the leaves, rich in phenolic components, showed increased GSH content at lower dose, increasing the dose further decreases the GSH content, an effect which may be due to cytotoxicity of high phenolic content. The erythrocyte membrane is prone to lipid peroxidation under oxidative stress that leads to the formation of MDA, a biomarker used for studying the oxidation of lipids under different conditions [59, 60]. Like GSH content, a similar pattern was observed for MDA, aqueous extract of *Moringa* leaves maintained the normal level of erythrocyte MDA, suggesting that the extract may have a mixture of biomolecules with hydroxyl groups that prevent the abstraction of hydrogen atom from the double bond of lipid bilayer thereby avoiding the damage of lipid membrane. An inhibition of lipid peroxidation (LPO) was observed in a dose-dependent manner, but it was not very significant. Our results are concomitant with our previous report for the *in vitro *analysis of the plant extracts [44], wherein the normal level of malondialdehyde content of the erythrocyte was maintained. But aqueous extract of fruit showed a significant reduction (*P* = 0.0121 for unpaired *t*-test), in malondialdehyde content at dose of 0.1 mg/kg b/w. As previously reported [13, 61], the reducing power of bioactive compounds was associated with antioxidant activity. Thus, for the explication of the relationship between antioxidant effect and reducing power of the phenolics, the determination of reducing power was a necessity. We did a comparative analysis for the antioxidant properties of aqueous and ethanolic extracts of *Moringa* leaf and fruit and assess their capacity to reduce potassium ferricyanide and scavenge free radicals both *in vitro *and *in vivo*. The total phenolic content of the *Moringa* leaf and fruit extracts was determined in terms of gallic acid equivalence. Our observations are in agreement with the previous findings of Siddhuraju and Becker [13], where high content of phenolics in the ethanolic extract of leaves compared to aqueous extract was reported. In addition, our findings on comparative analysis depict higher amount of phenolics in fruit (pod). *In vivo *analysis with serum showed a significant pattern (*P* = 0.0053) similar to that of *in vitro *studies. The reducing power of all the extracts showed a concentration- and dose-dependent increase in absorbance (*P* < 0.0001); however, when compared, the fruit extract proved to be better in both water and ethanol (*P* < 0.0001). The aqueous extract appeared to be less effective compared to ethanolic extract. In general, the higher polyphenols extraction yield corresponds with the higher antioxidant activity, probably due to the combined action of the substances in variable concentrations and their high hydrogen atom donating abilities. Our findings are well correlated with the amount of phenolic constituents present in the respective extract. The phenolics present in *Moringa* fruit extract are able to terminate the radical chain reaction by converting free radicals to more stable products. The FRAP analysis shows aqueous extract of *Moringa* fruit was the most effective (*P* < 0.0001). Similarly, a linear correlation between DPPH radical scavenging activity (*P* < 0.0001) and polyphenolic extract has been reported with variability in concentration and doses for the leaf extract [13].

The total antioxidant capacity was analysed in terms of ascorbic acid and was best observed at a concentration of 10 mg/kg b/w dose in leaf aqueous extract with significant *P* value of 0.05 which decreases on further increase in the dose. The antioxidant capacity of the ethanolic extract of fruit showed an increase with increasing dose from 0.01 mg/kg b/w to 100 mg/kg b/w (*P* < 0.05), which may be attributed to the high content of flavonoids such as kaempferol and other polyphenols [62]. Other compounds that might contribute to total antioxidant capacity are carotenoids and cinnamic acid derivatives [63]. Although total antioxidant capacity measurement does not represent the sum of activities of single antioxidants, it can be of clinical use, because it is an easy and less time consuming procedure.

To evaluate the toxicity of the extracts, doses of various concentrations ranging from 0.01 mg/kg b/w to 100 mg/kg b/w were analysed for toxicity. No mortality or morbidity was observed throughout the experimental period. Nonsignificant changes were observed in absolute weight and similarly no changes were observed in vital organ weight measurement both in absolute and relative terms. Haematological parameters showed nonsignificant changes except in RBC count at administration of ethanolic extract of *Moringa* leaves, but this was comparable and nonsignificant relative to the control system. Most of the serum biochemical parameters as SGPT, SGOT, and ALKP exhibited nonsignificant changes. The extracts were well tolerated till dose of 100 mg/kg b/w, with no toxicity.

Recently, we have showed concentration-dependent hydroxyl radical scavenging ability of *Moringa oleifera* fruit and leaf extracts in deoxy ribose degradation assay [64] and our present findings may in part not only suggest the use of *Moringa* fruit and leaves as a supplementary/dietary antioxidant in nutraceutical and/or cosmoceutical, but also improve the ethnopharmacological knowledge of *Moringa* plant, which paves the way for use of fruit and leaves as an economically viable source of natural and potent antioxidant.

## 5. Conclusion

Based on our observations, the ethanolic and aqueous extract of *Moringa* fruit and leaf significantly maintains the basal levels of GSH and MDA content in a concentration- and dose-dependent manner. The ethanolic extract of fruit showed highest phenolic content along with strong reducing power and free radical scavenging capacity. Safety evaluation studies showed that ethanolic and aqueous extract of both fruit and leaf was well tolerated by experimental animals. A high positive correlation was observed among the *in vitro *and *in vivo *assays for antioxidative properties. In addition, our results support the potent antioxidant activity of aqueous and ethanolic extract of *Moringa* which adds one more positive attribute to its known pharmacological properties and hence its use in traditional system of medicine. Further investigations on isolation, characterization, and identification of active phytoconstituents responsible for the protection of oxidative stress and antioxidant activity are warranted for future work.

## Supplementary Material

Table 1. The potential therapeutic values of Moringa oleifera against cancer, diabetes, rheumatoid arthritis and other diseases are due to the phytochemicals present in the various parts of the tree. Glucosinolates, carbamates and isothiocyanates are fairly unique group of compounds peculiar to Moringaceae family. Other metabolites include flavonoids, polysaccharide and sterols. The distribution of various phytochemicals varies with the plant part which gives either broad or specific bioactivity.
Table 2. The antioxidant capacity of the Moringa fruit (pod) is due to the presence of higher amount of isothiocyanates and other flavonoids compared to leaf.
Figures. The acute toxicity of ascorbic acid (standard antioxidant) and gallic acid (ubiquitously present in plant as phenolics) was also performed along with Moringa extracts for comparative analysis. Non-significant changes were observed for the body weight, biochemical and haematological parameters. The molecules were well tolerated and non toxic upto a checked dose of 10 mg/kg body weight.

## Abbreviations

ALKP: Alkaline Phosphatase
DMSO: Dimethyl sulfoxide
DPPH: 2, 2-diphenyl-2-picrylhydrazyl
DTNB: 5, 5′-Dithiobis 2-nitro benzoic acid
FRAP: Ferric reducing antioxidant power
GAE: Gallic acid equivalent
AAE: Ascorbic acid equivalent GSH, glutathione
GSH: Glutathione
MDA: Malondialdehyde
MFW: *Moringa* fruit (pod) aqueous extract
MFE: *Moringa* fruit (pod) ethanol extract
MLW: *Moringa* leaf aqueous extract
MLE: *Moringa* leaf ethanol extract
RP: Reducing power
Hb: Hemoglobin
RBC: Red blood corpuscles
WBC: White blood corpuscles
SGPT: Serum glutamic pyruvic transaminase
SGOT: Serum glutamic oxaloacetic transaminase
TAC: Total antioxidant capacity
t-BHP: Tertiary-Butyl hydroperoxide
TBA: Thiobarbituric acid
TCA: Trichloroacetic acid
TPC: Total phenolic content
TPTZ: Tripyridyl-s-triazine.

## References

[1] Somali MA, Bajneid MA, Al-Fhaimani SS (1984). Chemical composition and characteristics of *Moringa peregrina* seeds and seeds oil. *Journal of the American Oil Chemists’ Society*.

[2] Mughal MHS, Ali G, Srivastava PS, Iqbal M (1999). Improvement of drumstick (*Moringa pterygosperma* Gaertn.) A unique source of food and medicine through tissue culture. *Hamdard Medicus*.

[3] Morton JF (1991). The horseradish tree, *Moringa pterygosperma* (*Moringaceae*)-A boon to Arid Lands?. *Economic Botany*.

[4] Souza JD, Kulkarni AR (1993). Comparative studies on nutritive values of tender foliage of seedlings and mature plants of *Moringa oleifera* Lam. *Journal of Economic and Taxonomic Botany*.

[5] Palada MC (1996). *Moringa* (*Moringa oleifera* Lam.): a versatile tree crop with horticultural potential in the subtropical United States. *HortScience*.

[6] Fuglie LJ (1999). *The Miracle Tree: Moringa oleifera: Natural Nutrition for the Tropics*.

[7] Dillard CJ, Bruce German J (2000). Phytochemicals: nutraceuticals and human health. *Journal of the Science of Food and Agriculture*.

[8] Anwar F, Bhanger MI (2003). Analytical characterization of *Moringa oleifera* seed oil grown in temperate regions of Pakistan. *Journal of Agricultural and Food Chemistry*.

[9] Anwar F, Ashraf M, Bhanger MI (2005). Interprovenance variation in the composition of *Moringa oleifera* oilseeds from Pakistan. *JAOCS, Journal of the American Oil Chemists’ Society*.

[10] Anwar F, Latif S, Ashraf M, Gilani AH (2007). *Moringa oleifera*: a food plant with multiple medicinal uses. *Phytotherapy Research*.

[11] Thurber MD, Fahey JW (2009). Adoption of Moringa oleifera to combat under-nutrition viewed through the lens of the “Diffusion of innovations” theory. *Ecology of Food and Nutrition*.

[12] Fahey JW (2005). *Moringa oleifera*: a Review of the medical evidence for its nutritional, therapeutic, and prophylactic properties. Part 1. *Trees for Life Journal*.

[13] Siddhuraju P, Becker K (2003). Antioxidant properties of various solvent extracts of total phenolic constituents from three different agroclimatic origins of drumstick tree (*Moringa oleifera* Lam.) leaves. *Journal of Agricultural and Food Chemistry*.

[14] Estrella MCP, Mantaring JBV, David GZ (2000). A double blind randomised controlled trial on the use of malunggay (*Moringa oleifera*) for augmentation of the volume of breastmilk among non-nursing mothers of preterm infants. *The Philippine Journal of Pediatrics*.

[15] Pal SK, Mukherjee PK, Saha BP (1995). Studies on the antiulcer activity of *Moringa oleifera* leaf extract on gastric ulcer models in rats. *Phytotherapy Research*.

[16] Pal SK, Mukherjee PK, Saha K, Pal M, Saha BP (1995). Antimicrobial action of the leaf extract of *Moringa oleifera* Lam.. *Ancient Science of Life*.

[17] Tahiliani P, Kar A (2000). Role of *Moringa oleifera* leaf extract in the regulation of thyroid hormone status in adult male and female rats. *Pharmacological Research*.

[18] Dahot MU (1988). Vitamin contents of flowers and seeds of *Moringa oleifera*. *Pakistan Journal of Biochemistry*.

[19] Caceres A, Lopez S (1991). Pharmacological properties of *Moringa oleifera*. 3. Effect of seed extracts in the treatment of experimental pyodermia. *Fitoterapia*.

[20] Caceres A, Saravia A, Rizzo S, Zabala L, De Leon E, Nave F (1992). Pharmacologic properties of *Moringa oleifera*. 2: screening for antispasmodic, antiinflammatory and diuretic activity. *Journal of Ethnopharmacology*.

[21] Gilani AH, Aftab K, Shaheen F, Capasso F, Mascolo N (1992). Antispasmodic activity of active principle from *Moringa oleifera*. *Natural Drugs and the Digestive Tract*.

[22] Faizi S, Siddiqui BS, Saleem R, Siddiqui S, Aftab K, Gilani AUH (1994). Novel hypotensive agents, niazimin A, niazimin B, niazicin A and niazicin B from *Moringa oleifera*: isolation of first naturally occurring carbamates. *Journal of the Chemical Society, Perkin Transactions*.

[23] Faizi S, Siddiqui BS, Saleem R, Siddiqui S, Aftab K, Gilani AUH (1994). Isolation and structure elucidation of new nitrile and mustard oil glycosides from *Moringa oleifera* and their effect on blood pressure. *Journal of Natural Products*.

[24] Faizi S, Siddiqui BS, Saleem R, Aftab K, Shaheen F, Gilani AUH (1998). Hypotensive constituents from the pods of *Moringa oleifera*. *Planta Medica*.

[25] Faizi S, Siddiqui BS, Saleem R, Siddiqui S, Aftab K, Gilani AH (1995). Fully acetylated carbamate and hypotensive thiocarbamate glycosides from *Moringa oleifera*. *Phytochemistry*.

[26] Ghasi S, Nwobodo E, Ofili JO (2000). Hypocholesterolemic effects of crude extract of leaf of *Moringa oleifera* Lam in high-fat diet fed wistar rats. *Journal of Ethnopharmacology*.

[27] Dangi SY, Jolly CI, Narayanan S (2002). Antihypertensive activity of the total alkaloids from the leaves of *Moringa oleifera*. *Pharmaceutical Biology*.

[28] Mehta LK, Balaraman R, Amin AH, Bafna PA, Gulati OD (2003). Effect of fruits of *Moringa oleifera* on the lipid profile of normal and hypercholesterolaemic rabbits. *Journal of Ethnopharmacology*.

[29] Gilani AH, Aftab K, Suria A (1994). Pharmacological studies on hypotensive and spasmolytic activities of pure compounds from *Moringa oleifera*. *Phytotherapy Research*.

[30] Das BR, Kurup PA, Rao PL (1957). Antibiotic principle from *Moringa pterygosperma*. VII. Antibacterial activity and chemical structure of compounds related to pterygospermin. *The Indian Journal of Medical Research*.

[31] Eilert U, Wolters B, Nahrstedt A (1981). The antibiotic principle of seeds of *Moringa oleifera and Moringa Stenopetala*. *Planta Medica*.

[32] Caceres A, Cabrera O, Morales O, Mollinedo P, Mendia P (1991). Pharmacological properties of *Moringa oleifera*. 1: preliminary screening for antimicrobial activity. *Journal of Ethnopharmacology*.

[33] Nikkon F, Saud ZA, Rehman MH, Haque ME (2003). In vitro antimicrobial activity of the compound isolated from chloroform extract of *Moringa oleifera* Lam.. *Pakistan Journal of Biological Sciences*.

[34] Murakami A, Kitazono Y, Jiwajinda S, Koshimizu K, Ohigashi H (1998). Niaziminin, a thiocarbamate from the leaves of *Moringa oleifera*, holds a strict structural requirement for inhibition of tumor-promoter-induced epstein- barr virus activation. *Planta Medica*.

[35] Guevara AP, Vargas C, Sakurai H (1999). An antitumor promoter from *Moringa oleifera* Lam. *Mutation Research*.

[36] Bharali R, Tabassum J, Azad MR (2003). Chemomodulatory effect of *Moringa oleifera*, Lam, on hepatic carcinogen metabolising enzymes, antioxidant parameters and skin papillomagenesis in mice. *Asian Pacific Journal of Cancer Prevention*.

[37] Gilani AH, Janbaz KH, Shah BH (1997). Quercetin exhibits hepatoprotective activity in rats. *Biochemical Society Transactions*.

[38] Ndabigengesere A, Subba Narasiah K, Talbot BG (1995). Active agents and mechanism of coagulation of turbid waters using *Moringa oleifera*. *Water Research*.

[39] Ghebremichael KA, Gunaratna KR, Henriksson H, Brumer H, Dalhammar G (2005). A simple purification and activity assay of the coagulant protein from *Moringa oleifera* seed. *Water Research*.

[40] Oliveira JTA, Silveira SB, Vasconcelos IM, Cavada BS, Moreira RA (1999). Compositional and nutritional attributes of seeds from the multiple purpose tree *Moringa oleifera* Lamarck. *Journal of the Science of Food and Agriculture*.

[41] The Wealth of India (A Dictionary of Indian Raw Materials and Industrial Products) (1960). *Raw Materials*.

[42] Luqman S, Rizvi SI (2006). Protection of lipid peroxidation and carbonyl formation in proteins by capsaicin in human erythrocytes subjected to oxidative stress. *Phytotherapy Research*.

[43] Rizvi SI, Luqman S (2002). Anti-oxidative property of capsaicin. *Medicinal Chemistry Research*.

[44] Luqman S, Kaushik S, Srivastava S (2009). Protective effect of medicinal plant extracts on biomarkers of oxidative stress in erythrocytes. *Pharmaceutical Biology*.

[45] Singleton VL, Rossi JA (1965). Colorimetry of total phenolics with phosphomolybdic-phosphotungstic acid reagents. *American Journal of Enology and Viticulture*.

[46] Luqman S, Kumar R, Kaushik S, Srivastava S, Darokar MP, Khanuja SPS (2009). Antioxidant potential of the root of *Vetiveria zizanioides* (L.) Nash. *Indian Journal of Biochemistry and Biophysics*.

[47] Yen GC, Chen HY (1995). Antioxidant activity of various tea extracts in relation to their antimutagenicity. *Journal of Agricultural and Food Chemistry*.

[48] Benzie IFF, Strain JJ (1996). The ferric reducing ability of plasma (FRAP) as a measure of ’antioxidant power’: the FRAP assay. *Analytical Biochemistry*.

[49] Chung YC, Chang CT, Chao WW, Lin CF, Chou ST (2002). Antioxidative activity and safety of the 50% ethanolic extract from red bean fermented by Bacillus subtilis IMR-NK1. *Journal of Agricultural and Food Chemistry*.

[50] Prieto P, Pineda M, Aguilar M (1999). Spectrophotometric quantitation of antioxidant capacity through the formation of a phosphomolybdenum complex: specific application to the determination of vitamin E. *Analytical Biochemistry*.

[51] Beutler E, Duron O, Kelly BM (1963). Improved method for the determination of blood glutathione. *The Journal of Laboratory and Clinical Medicine*.

[52] Esterbauer H, Cheeseman KH (1990). Determination of aldehydic lipid peroxidation products: malonaldehyde and 4-hydroxynonenal. *Methods in Enzymology*.

[53] Joshua Allan J, Damodaran A, Deshmukh NS, Goudar KS, Amit A (2007). Safety evaluation of a standardized phytochemical composition extracted from *Bacopa monnieri* in Sprague-Dawley rats. *Food and Chemical Toxicology*.

[54] Chanda D, Shanker K, Pal A (2009). Safety evaluation of trikatu, a generic ayurvedic medicine in Charles Foster rats. *Journal of Toxicological Sciences*.

[55] Liu JY, Chen CC, Wang WH, Hsu JD, Yang MY, Wang CJ (2006). The protective effects of *Hibiscus sabdariffa* extract on CCl 4-induced liver fibrosis in rats. *Food and Chemical Toxicology*.

[56] Amin A, Hamza AA (2005). Hepatoprotective effects of *Hibiscus*, *Rosmarinus* and *Salvia* on azathioprine-induced toxicity in rats. *Life Sciences*.

[57] Reglinski J, Hoey S, Smith WE, Sturrock RD (1988). Cellular response to oxidative stress at sulfhydryl group receptor sites on the erythrocyte membrane. *The Journal of Biological Chemistry*.

[58] Di Simplicio P, Lupis E, Rossi R (1996). Different mechanisms of formation of glutathione-protein mixed disulfides of diamide and tert-butyl hydroperoxide in rat blood. *Biochimica et Biophysica Acta*.

[59] Konukoglu D, Akçay T, Erdemm T (1998). Susceptibility of erythrocyte lipids to oxidation and erythrocyte antioxidant status in myocardial infarction. *Clinical Biochemistry*.

[60] López-Revuelta A, Sánchez-Gallego JI, Hernández-Hernández A, Sánchez-Yagüe J, Llanillo M (2005). Increase in vulnerability to oxidative damage in cholesterol-modified erythrocytes exposed to *t*-BuOOH. *Biochimica et Biophysica Acta*.

[61] Yen GC (1993). Relationship between antioxidant activity and maturity of peanut hulls. *Journal of Agricultural and Food Chemistry*.

[62] Bennett RN, Mellon FA, Foidl N (2003). Profiling glucosinolates and phenolics in vegetative and reproductive tissues of the multi-purpose trees *Moringa oleifera* L. (Horseradish tree) and Moringa stenopetala L. *Journal of Agricultural and Food Chemistry*.

[63] Taga MS, Miller EE, Pratt DE (1984). Chia seeds as a source of natural lipid antioxidants. *Journal of the American Oil Chemists’ Society*.

[64] Luqman S, Kumar R (2011). Attenuation of hydroxyl radical formation by extracted constituent of *Moringa oleifera* lam.. *Current Chemical Biology*.

