# Supplementary material for: Experimental Assessment of Moringa oleifera Leaf and Fruit for Its Antistress, Antioxidant, and Scavenging Potential Using In Vitro and In Vivo Assays
Source: Evid Based Complement Alternat Med. 2011 Dec 14;2012:519084. doi: 10.1155/2012/519084 (PMC3247066; doi:10.1155/2012/519084)
Supplement: Supplementary file 1 — Table 1. The potential therapeutic values of Moringa oleifera against cancer, diabetes, rheumatoid arthritis and other diseases are due to the phytochemicals present in the various parts of the tree. Glucosinolates, carbamates and isothiocyanates are fairly unique group of compounds peculiar to Moringaceae family. Other metabolites include flavonoids, polysaccharide and sterols. The distribution of various phytochemicals varies with the plant part which gives either broad or specific bioactivity. Table 2. The antioxidant capacity of the Moringa fruit (pod) is due to the presence of higher amount of isothiocyanates and other flavonoids compared to leaf. Figures. The acute toxicity of ascorbic acid (standard antioxidant) and gallic acid (ubiquitously present in plant as phenolics) was also performed along with Moringa extracts for comparative analysis. Non-significant changes were observed for the body weight, biochemical and haematological parameters. The molecules were well tolerated and non toxic upto a checked dose of 10 mg/kg body weight. [file 519084.f1.pdf]

Supplementary Data:

Table 1. Constituents present in different parts of moringa tree.

**Parts**	**Phytochemical constituents**
Stem	octacosanic acid, 4-hydroxymellein, vanillin, β-sitosterone and β-sitosterol
Bark	4-(α-L-rhamnopyranosyloxy)-benzylglucosinolate
Whole gum exudates	L-arabinose, D-galactose, L-rhamnose, D-mannose, D-xylose, D-glucuronic acid and leucoanthocyanin
Leaves	Glycoside niazirin, niazirinin and three mustard oil glycosides, 4-[4'-O-acetyl- α -L-rhamnosyloxy) benzyl] isothiocyanate, niaziminin A and B
Mature flowers	D-mannose, D-glucose, protein, ascorbic acid, polysaccharide
Whole pods	Nitriles, isothiocyanate, thiocarbanates, 0-[2'-hydroxy-3'-(2''-heptenyloxy)]-propylundecanoate, 0-ethyl-4-[(α -1-rhamnosyloxy)-benzyl] carbamate, methyl-p-hydroxybenzoate and β-sitosterol
Mature seeds	Crude protein, Crude fat, carbohydrate, methionine, cysteine, 4-(α-L-rhamnopyranosyloxy)-benzylglucosinolate, benzylglucosinolate, moringyne, mono-palmitic and di-oleic triglyceride
Seed oil	Vitamin A, beta carotene, precursor of Vitamin A
Roots	4-(α-L-rhamnopyranosyloxy)-benzylglucosinolate and benzylglucosinolate

Ref: Pharmacognosy Reviews [Phcog Rev.] 2008 –Supplement Vol 2 (4), Page 7-13

Table 2. Different compounds present in leaves and pods of moringa

Leaves	Pods		
Niaziminin A	Pentacosane	4-(p-Hydroxy) Phenyl ethylbutanoate	9-Octadecenoic acid
Niaziminin B	Heptacosane	Isothiocyanato hexanoic acid	4, 10-Dimethyl-13-nonacosanol
4-(2’,3’,4’-Tri-Oacetyl-a-L-rhamnosyloxy) benzyl nitrile	Nonacosane	Octadecanoic acid	Docosanoic acid
Niazicinin A	Sulfur	Eicosanoic acid	15-Cyano-2-methyl pentadecanoic acid
Methyl-4-(2’,3’,4’-tri-O-acetyl-a-L-rhamnosyloxy) benzyl carbamate (E)	Methyl hexadecanoate	Tetracosanoic acid	9, 12-Octadecadienoic acid
Methyl-4-(2’,3’,4’-tri-O-acetyl-a-L-rhamnosyloxy) benzyl thiocarbamate (E)	Ethyl pentadecanoate	β-Sitosterol Propyl-p-hydroxy benzoate	4,11-Dimethyl-12-docosanol
Methyl-4-(2’,3’,4’-tri-O-acetyl-a-L-rhamnosyloxy) benzyl thiocarbamate (Z)	Ethyl hexadecanoate	Heptadecadien – 2 one	Methyl p-hydroxybenzoate
Methyl-4-(2’,3’,4’-tri-O-acetyl-a-L-rhamnosyloxy) benzyl carbamate (Z)	Ethyl heptadecanoate	6-Methyl docosane	11-Cabonyl-12, 16-dioxo-14-hydroxy-18-tricoasene
Niazimicin B	Methyl octadecanoate	Ethyl octadeca-9,12-dienoate	Ethy1-4(a-L-rhamnosyloxy) benzyl carbamate
Ethyl-4-(2’,3’,4’-tri-O-acetyl-a-L-rhamnosyloxy) benzyl carbamate (E)	Ethyl octadecanoate	Docoasen-8-ol	p-Hydroxybenzyl ethyl carbamate
Niazicin B	Ethyl eicosanoae	p-Hydroxyphenyl methoxyethance	Tetradecanoic acid
O-Ethyl-4 (2’,3’,4’-tri-O-acetyl-a-L-rhamnosyloxy) benzy1 thiocarbamate (Z)	Ethyl docosanoate	6, 9-Dimethyl dodecanoic acid	Heptadecanoic acid
Niazinin B	Ethyl 9-octadecenoate	8-Heptadecanol	Dodecanyl isothiocyanate
Niazimicin A	Ethyl tricosanoate	8-Nonadecanol	9-Methyl-eicoasen-1, 11-diol
Niazinin A	Ethyl-9-hexadecenoate	9-Methyl pentadecene isothiocyanate	p-Aminobenzaldehyde
Niazirinin	Methyl octadeca-9, 12-dienoate	Hexadecanoic acid	p-Hydroxyphenyl acetonitrile
NiaziminB	Ethyl heptadeca-9, 12-dienoate	6-Methyl eicosane	2-Octadecanone
4 (4’-O-Acetyl-a-L-rhamnosyloxy) benzaldehyde	Ethyl 9-nonadecenoate	1-Ethoxytricontane	Methyl 9-octadecenoate
Niazimin A	9-Methyl octadecane nitrile	1-Ethoxyheptadecane	Tetradecanyl isothiocyanate
Niazicin A	Isothiocyanato-4-hexenoic acid	Heptadecane nitrile	1-Ethoxypentadecane
4-(4’-O-Acetyl-a-Lrhamnosyloxy) benzyl isothiocyanate	Isothiocyanato-3-pentenoic acid	10-Methyl nonadecane isocyanate	Heptadecane isocyanate
Niazirin	7-(p-Hydroxy) phenoxy heptanoic acid	Tridecene	Octadecane isocyanate
4-(a-L-Rhamnosyloxy) benzyl isthiocyanate	(p-Hydroxy) Phenoxy acetic acid	p-Hydroxybenzoic acid	Tricosane isothiocyanate

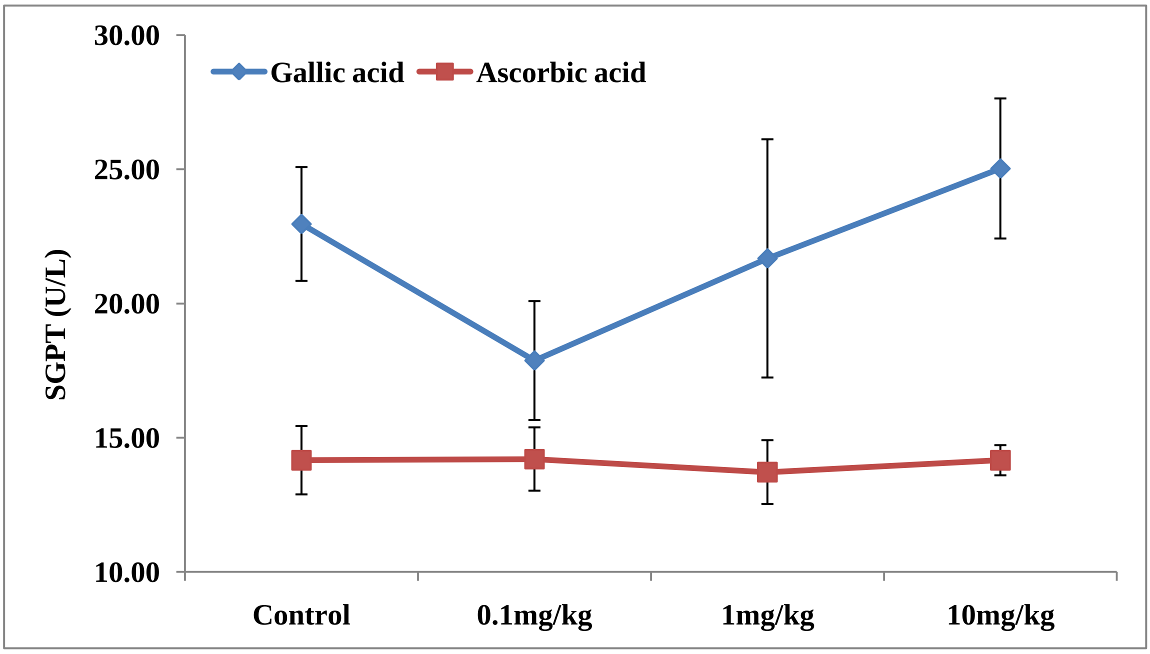
30.00
25.00
20.00
15.00
10.00
SGPT (U/L)
Gallic acid
Ascorbic acid
Control
0.1mg/kg
1mg/kg
10mg/kg

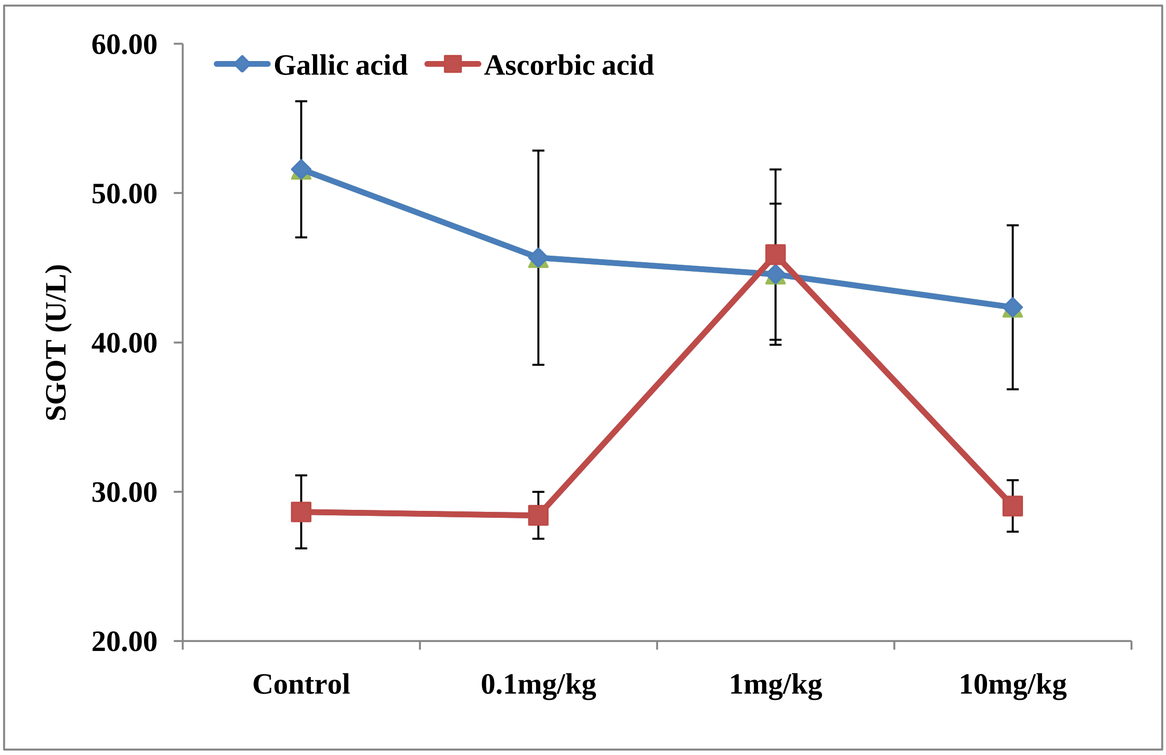
60.00
50.00
40.00
30.00
20.00
SGOT (U/L)
Gallic acid
Ascorbic acid
Control
0.1mg/kg
1mg/kg
10mg/kg

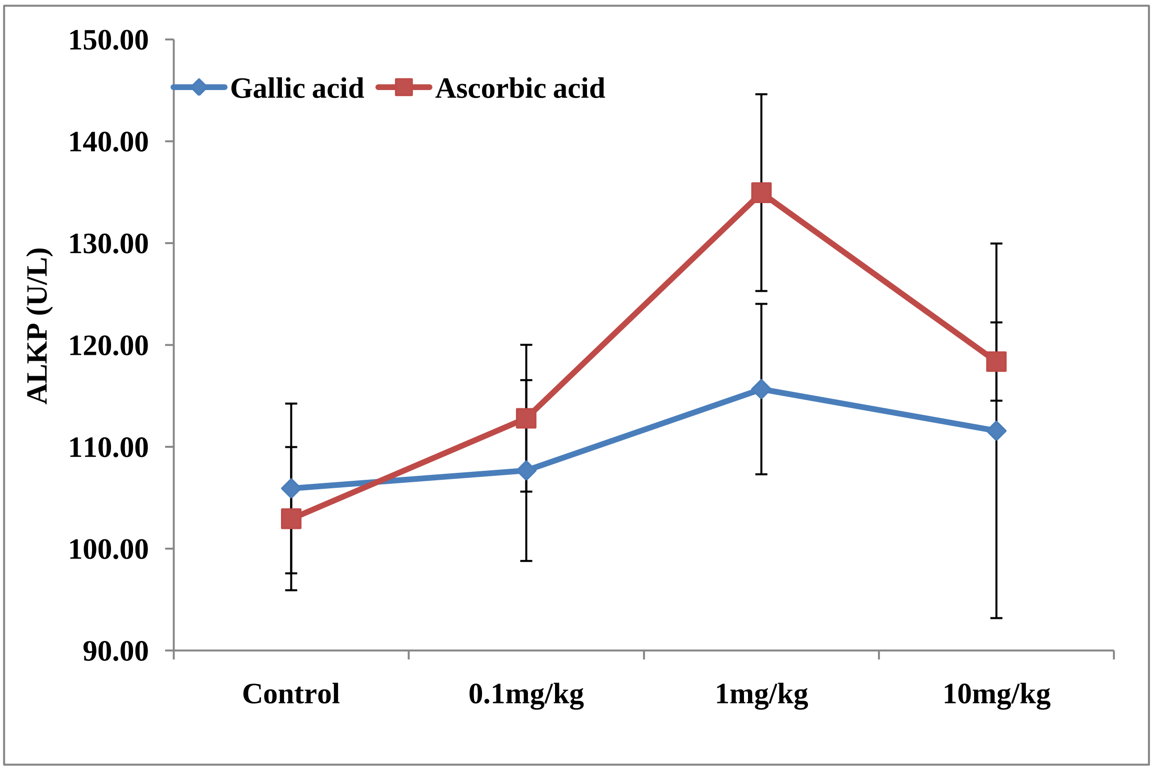
150.00
140.00
130.00
120.00
110.00
100.00
90.00
ALKP (U/L)
Gallic acid
Ascorbic acid
Control
0.1mg/kg
1mg/kg
10mg/kg

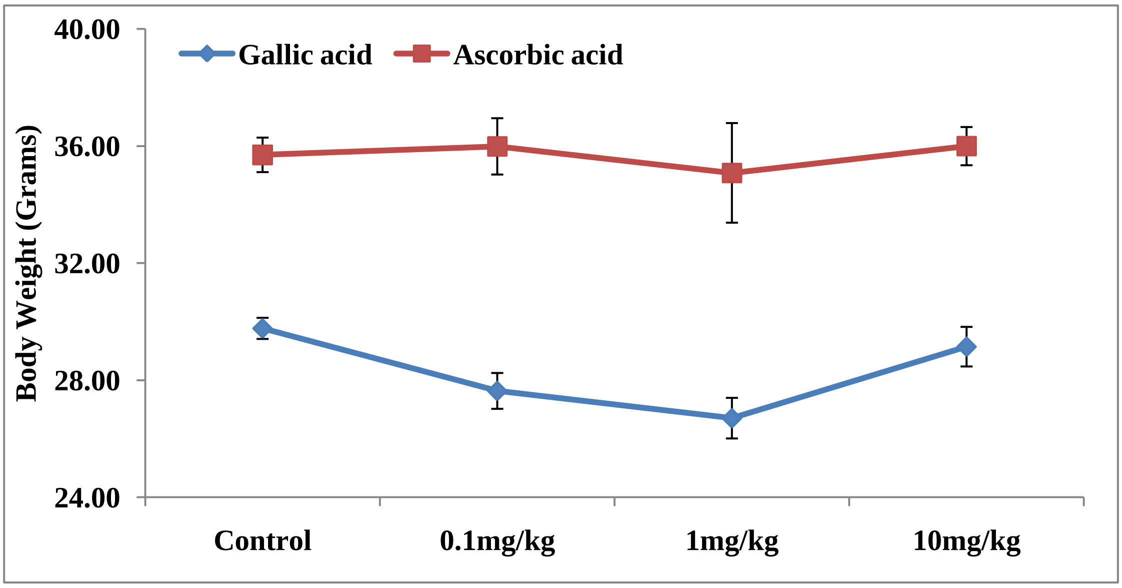
40.00
36.00
32.00
28.00
24.00
Body Weight (Grams)
Gallic acid
Ascorbic acid
Control
0.1mg/kg
1mg/kg
10mg/kg
